# Cell-Based Regenerative Strategies for Treatment of Diabetic Skin Wounds, a Comparative Study between Human Umbilical Cord Blood-Mononuclear Cells and Calves' Blood Haemodialysate

**DOI:** 10.1371/journal.pone.0089853

**Published:** 2014-03-18

**Authors:** Hala O. El-Mesallamy, Mohamed R. Diab, Nadia M. Hamdy, Sarah M. Dardir

**Affiliations:** 1 Biochemistry Department, Faculty of Pharmacy, Ain-Shams University, Cairo, Egypt; 2 Research Department, Holding Company for Biological Products and Vaccines (VACSERA), Giza, Egypt; Sungkyunkwan University, Korea, Republic of

## Abstract

**Background:**

Diabetes-related foot problems are bound to increase. However, medical therapies for wound care are limited; therefore, the need for development of new treatment modalities to improve wound healing in diabetic patients is essential and constitutes an emerging field of investigation.

**Methods:**

Animals were randomly divided into 8 groups (I–VIII) (32 rats/group), all were streptozotocin (STZ)-induced diabetics except groups III and VIII were non-diabetic controls. The study comprised two experiments; the first included 3 groups. Group I injected with mononuclear cells (MNCs) derived from human umbilical cord blood (HUCB), group II a diabetic control group (PBS i.v). The second experiment included 5 groups, groups IV, V, and VI received topical HUCB-haemodialysate (HD), calves' blood HD, and solcoseryl, respectively. Group VII was the diabetic control group (topical saline). Standard circular wounds were created on the back of rats. A sample of each type of HD was analyzed using the high performance liquid chromatography-electrospray ionization-mass spectrometry (HPLC-ESI-MS) system. Wound area measurement and photography were carried out every 4 days. Plasma glucose, catalase (CAT), malondialdehyde (MDA), nitric oxide (NO) and platelets count were assessed. Wound samples were excised for hydroxyproline (HP) and histopathological study.

**Results:**

Treatment with HUCB MNCs or HUCB-HD resulted in wound contraction, increased CAT, NO, platelets count, body weights, and HP content, and decreased MDA and glucose.

**Conclusion:**

Systemic administration of HUCB MNCs and topical application of the newly prepared HUCB-HD or calves' blood HD significantly accelerated the rate of diabetic wound healing and would open the possibility of their future use in regenerative medicine.

## Introduction

From the chronic complications of diabetes are neuropathy and variety of connective tissue abnormalities [1]. Foot ulceration affects (15–25%) of all diabetic patients during their lifetime [2]. Only 2/3 of diabetic foot ulcers heal and up to 28% may result in lower extremity amputation [3]. To prevent or reduce surgical intervention, new therapeutic strategies are to be developed to improve diabetic wound healing.

Cell therapy is a promising approach for treating diabetic non-healing wounds [4]. The aim of cell-based regenerative strategies is repair or enhancement of the damaged tissues' biological function, by utilizing cells and/or bioactive molecules [5]. This can be carried out by transplantation, through local delivery or systemic infusion of autologous or allogenic cells [6]. These cells include primary cells, cell lines, and various stem cells [5]. Among the main sources of stem cells that might be used for regeneration of injured skin tissue are adult stem cells and embryonic stem cells (ESCs). ESCs have great capacity for self-renewal and pluripotency, but their clinical applications that are associated with ethical and legal issues have shifted the focus to adult stem cells [7].

A population of adult stem cells especially mesenchymal stem cells (MSC) resides within most of adult mammalian tissues/organs, and the most common sources include bone marrow and umbilical cord blood [5]. Bone marrow (BM) BM-MSC can produce multiple types of skin cell and insulin expressing cells [8], but the source of BM is limited and their capacity for differentiation decline with age [9]. Human umbilical cord blood (HUCB) as a source of stem cells is readily available, on non-invasive collection and can be routinely harvested without any risk for the donor babies [10]. HUCB contains stem cells in greater number than BM [11]. The incidence of graft-versus host disease (GVHD) is lower in HUCB transplantation than other allogenic cell-based therapies. Therefore, the application of HUCB cell turned out to be an excellent alternative source of haemopoietic stem cells to other allogenic cell-based therapies [12].

Haemodialysate (HD) is another approach that has been developed a few decades ago; to improve situations of impaired healing in both experimental and human approaches [13]. Solcoseryl, referred to as the commercially available HD, is a chemically and biologically standardized, protein free, non pyrogenic, and non antigenic dialysate derived from healthy suckling calves' blood [14]. Solcoseryl activity is ascribed to its constituents, being a broad spectrum of natural low molecular weight substances including electrolytes, amino acids, lipids, phospholipids, essential trace element, and intermediate products of carbohydrate and fat metabolism [15]. Hence, the aim of the present study is to evaluate MNCs derived from HUCB as a cell therapy for diabetic wounds compared to untreated diabetic and normal wounds. In addition, HUCB will be used in a different way to prepare a low molecular weight fraction; “HD” which will be investigated as a potential topical treatment for the same condition in comparison to a HD prepared from calves' blood, and the commercially available product; “solcoseryl”. Knowing that this type of HD has never been prepared before from HUCB, and has never been used in an experimental study focusing on diabetic wounds, we believe that this study hopefully, provides an innovation in diabetic wound treatment.

## Materials and Methods

### 1. Ethics statement

Animals were housed in accordance with the Principles of laboratory animal care (NIH publication no. 85–23, revised 1985; http://grants1.nih.gov/grants/olaw/references/phspol.htm). The experimental protocol was approved as well by Faculty of Pharmacy, Ain Shams University/VACSERA ethical committees. An informed written consent was obtained from each mother participating in the study after approval of the General Organization for Teaching Hospitals & Institutes (GOTHI) research ethics committee as well as Faculty of Pharmacy, Ain Shams University/VACSERA ethical committees.

### 2. Drugs and chemicals

Solcoseryl ampoules were obtained from Misr Co. for Pharm. Ind. S.A.E under license of Valeant Pharmaceuticals Co. Switzerland GMBH. Other biochemical reagents, unless otherwise specified, were purchased from Sigma-Aldrich Chemical Co. (St. Louis, Mo., USA).

### 3. Animals

The study was performed on 256 adult male Albino rats of Wistar strain weighing (150–200 gm), obtained from the breeding colony of Helwan farm belonging to the Holding company for biological products and vaccines (VACSERA, Egypt).

### 4. Experimental design

Animals were randomly divided into 8 groups (I–VIII) (32 rats/group), all were diabetic except groups III and VIII were non-diabetic control groups.

### 5. Experimental induction of diabetes

Rats were made diabetic by a single I.P injection of 50 mg/kg body weight of STZ dissolved in citrate buffer (0.01 mol/l, pH 4.5). Rats serving as controls were given the same volume of sodium citrate. Diabetes was confirmed by determination of fasting blood glucose (FBG) concentration 3-days post STZ injection showing FBG levels above 250 mg/dl [16].

### 6. Collection of HUCB and isolation of cells

HUCB samples were collected from healthy full-term (36–40 weeks) normal human deliveries [17]; they were obtained from the Department of Obstetrics and Gynecology, El-Galaa hospital, Cairo, Egypt. Each cord blood sample was collected into a 50 ml sterile polypropylene tube containing 5 ml citrate phosphate dextrose (CPD) anticoagulant [18].To isolate MNCs, each UCB unit was carefully layered onto Ficoll separating solution (BIOCHROM AG, Berlin, Germany) (density = 1.077 g/ml) inside a disposable 15 ml centrifuge tube. After density gradient centrifugation at 400×g (∼1500 rpm) for exactly 30 min at room temperature, the MNCs layer was removed from the interface, washed twice in PBS and centrifuged for 10 min at 250×g (∼1200 rpm) [19]. Cell viability was more than 95% by Trypan-Blue exclusion. The number of cells was optimized to be (400×10^6^) viable MNCs suspended in 0.5 ml PBS and injected one time into each animal via the tail vein (slow I.V injection).

### 7. Preparation of HUCB or calves' blood HD

Blood was applied to an Amicon device (Millipore Corporation-model, 8200, 200 ml, MA, USA) stirred ultra filtration cell containing nitrocellulose membrane of cut-off 10,000 Dalton. The system was surrounded with ice and put on a magnetic stirrer during the whole run (to avoid denaturation or decomposition of the blood components by the action of heat). Ultrafilterated blood containing the very low molecular weight substances was collected in sterile tubes and known as “HD”. The produced HD was then filtered through sterile non-pyrogenic filter units (Schleicher & Schuell) (pore size = 0.2 µm) and aliquoted into small tubes, a sample of which was analyzed for its contents using (HPLC-ESI-MS) at the National Research Center (Dokki, Giza, Egypt) [20] and the remaining aliquots were stored inside a deep freezer (−70°C) until used.

### 8. Acute toxicity (LD50) study

Initially, a dose–response study was executed for selecting HD dose. Tested or prepared drug (calf or human UCB-HD) was injected separately in a group of mice weighing 20–25 g I.P at 0.4, 0.5, 0.6, 0.7, 0.8, 0.9, 1.25, 1.5, 1.75, and 2 ml doses per 20 g of mice, where each dose was given to 6 mice/cage. All mice were subjected to careful observation for any toxic symptoms or deaths for 3 consecutive days (72 h) [21]. This was followed by tissue culture for the determination of the minimum effective therapeutic dose of both types of HD [22].

### 9. Induction of skin ulcer (Excision wound creation)

All rats (diabetic and non-diabetic) were anesthetized with diethyl ether. The hair of each rat was shaved with an electric clipper. Area of the wound to be created was outlined on the dorsal skin with a marking pen and a template (1.5 cm diameter) just before skin excision [23].The entire wound was left open.

### 10. Evaluation of wound healing

#### 10.1. Wound area measurement & photography

Starting from the day of wound induction (defined as day 0) and then on days 4, 8, 12, 16, and 20 post-wounding, wounds were photographed using a Canon (Canon PowerShot A2000 IS, Japan) digital camera and wound margins were traced on transparency papers then subsequently transferred to 1 mm^2^ graph sheets from which the wound surface area was evaluated. Percentage of wound contraction was calculated using the following formula: [(Initial wound size- specific day wound size)/Initial wound size]×100 [24]. Period of epithelialization was defined as the number of days taken for the complete healing of wounds so that no raw wound is left behind [25].

#### 10.2. Experimental protocol

The first experiment comprised investigation of the healing effect of undifferentiated MNCs isolated from HUCB on diabetic ulcer. Groups I and II were STZ-induced diabetic. Group I designated as HUCB MNCs group; was treated with (400×10^6^) viable MNCs suspended in 0.5 ml PBS (HUCB MNCs) and injected one time slowly into each animal via the tail vein immediately after wound creation. Group II was a diabetic control group and was injected with PBS i.v. The third group served as a normal (non-diabetic) control group, receiving no treatment.

The second experiment was designed to investigate the effect of cord blood and Calf blood HD on diabetic ulcers healing in comparison to solcoseryl (positive control drug). Groups IV, V, VI, and VII were STZ-induced diabetic and were topically treated with 150 µL of the treatment agent. Groups IV and V received prepared HUCB-HD and calves' blood HD, respectively. However, group VI served as a positive control group and was treated with solcoseryl. Group VII was a diabetic control group, which received normal saline, while the last group (VIII) was a non-diabetic control group receiving no treatment.

### 11. Biochemical analysis

Pre and post- treatment, the animals' body weights and FBG were determined. Blood samples for performing different biochemical and haematological tests were obtained by retro-orbital venous plexus puncture and collected into tubes containing the type of anticoagulant suitable for each test. Fluoride oxalate for determination of FBG [26], EDTA for determination of platelets count using an automated haematology analyzer (Cell-Dyn Emerald, Abbott Diagnostics, France) and plasma NO [27], sodium citrate for determination of MDA by thiobarbituric acid reactive species method [28] and CAT [29] by enzymatic kits supplied by Biodiagnostics (Giza, Egypt). Wound tissue samples were removed for the determination of granulation tissue weight, and stored at −70°C for subsequent biochemical assay of hydroxyproline (HP) [30] as an indicator of total collagen content.

Representative wound tissue from each group were fixed in 10% formol saline for subsequent histopathological examination by hematoxylin and eosin (H&E) [31].

### 12. Statistical analysis

Data are presented as means ± SD. Multiple group comparisons were carried out using one-way analysis of variance (ANOVA) followed by the Tukey-Kramer test for post hoc analysis. Statistical significance was acceptable to a level of p≤0.05. Data analysis was performed using the Statistical Package for Social Sciences (SPSS) version 19 software package, IBM Corp., USA, 2010).

## Results

**Figure 1:**
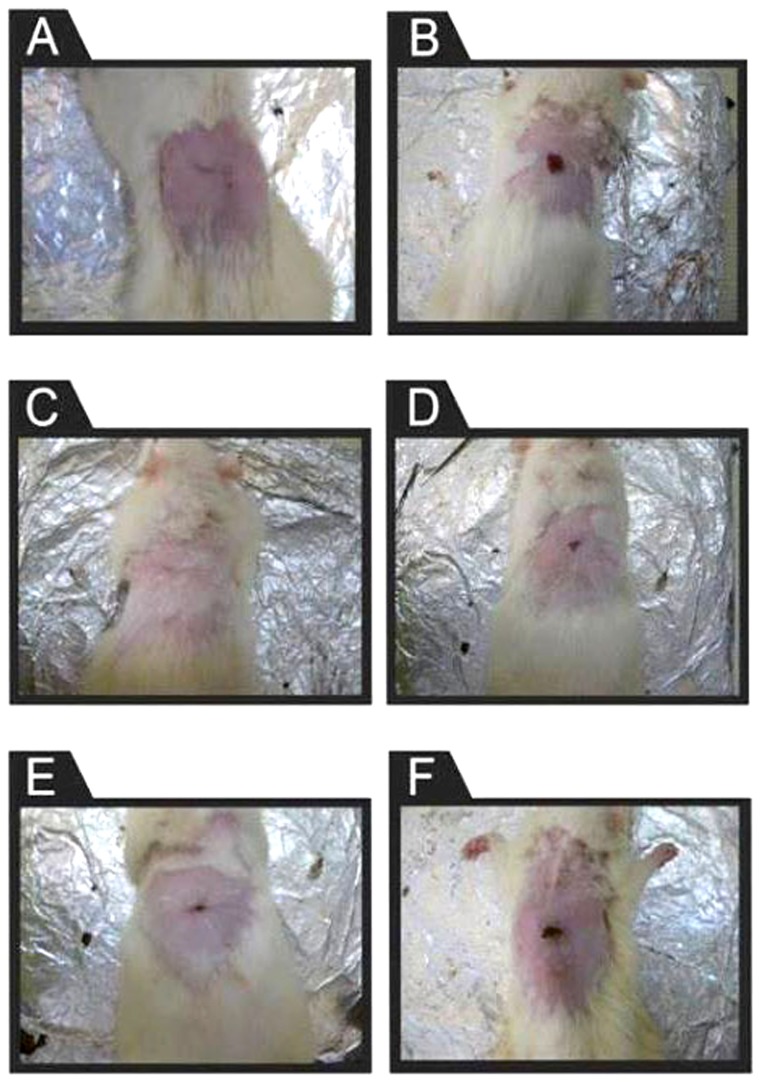
Gross appearance of wound healing on day 16 post-wounding. (a) diabetic wound treated with HUCB MNCs i.v, (b) diabetic control group (PBS i.v) (c) diabetic wound treated topically with HUCB-HD, (d) diabetic wound treated topically with calves' blood HD, (e) positive control group (diabetic wound) treated topically with solcoseryl, (f) diabetic control group (topical saline).

Only after 4 days of treatment, HUCB MNCs resulted in a significant (p≤0.001) wound closure accounting for about half of the originally opened area of the wound (49.19%) as compared to both diabetic (18.19%) and non diabetic control groups (17.4%). Closure of wounds continued significantly for this group until reaching (98.38%) on day 12 post-wounding and was almost complete (99.46%) by reaching day 16 post-wounding (**Table 1**) (**Figure 1A, B**). HUCB-HD-treated group showed the highest initial closure percentage (50.8%) among all groups. Only (0.02±0.02 cm^2^) of the original wound area was still open on day 12 post-wounding, and it was the first group to attain complete healing (100% closure) before day 16. Whereas on day 16, the calves' blood HD group was about to heal (99.46%), followed by the solcoseryl group (96.24%) which was close to the normal control group (96.74%). The diabetic control group had a still open wound by (0.12±0.04 cm^2^) (**Table 1**) (**Figure 1C, D, E, F**).

**Table 1:** Wound area, period of epithelialization, wet and dry granulation wt and hydroxyproline of treated and untreated diabetic and normal rats in the different studied groups.

Experiment	1^st^ Experiment (i.v)			2^nd^ Experiment (topical)				
Parameter	HUCB MNCs	Diabetic Control (PBS)	Control (non-diabetic)	HUCB-HD	Calves' blood HD	Solcoseryl	Diabetic control(saline)	Control (non-diabetic)
**Wound area(cm^2^)**								
Day 0	1.85±0.2	1.87±0.09	1.85±0.1	1.87±0.1	1.85±0.1	1.85±0.1	1.86±0.1	1.85±0.1
Day 4	0.94±0.2^a^	1.53±0.1	1.52±0.1	0.92±0.3^b^	1.35±0.1	1.47±0.3	1.52±0.3	1.52±0.1
Day 8	0.43±0.1^a^	1.36±0.08	1.15±0.2	0.3±0.1^b^	0.69±0.3^b^	0.96±0.3	1.18±0.2	1.15±0.2
Day 12	0.03±0.03^a^	0.87±0.3	0.56±0.1	0.02±0.02^b^	0.12±0.1^b^	0.4±0.3	0.54±0.4	0.56±0.1
Day 16	0.01±0.01^a^	0.6±0.4	0.04±0.02	0±0	0.01±0.02	0.07±0.1	0.12±0.04	0.04±0.02
Day 20	0±0	0.31±0.4	0.006±0.008	0±0	0±0	0.002±0.004	0.04±0.04	0.006±0.008
**Epithelialization(days)**	15.7 ±1.2^a^	22.8±2.2	19.7±1.8	13.5±1.4^b^	16.8 ±1.8^b^	18.2±2.3^b^	23.5 ±2.1	19.7±1.8
**Wet granulation wt (mg/100 g rat)**	163±7.2^a^	110±6.3	120±3.5	168±10^b^	130±2.2^b^	119±2.7	113±2.7	120±3.5
**Dry granulation wt (mg/100 g rat)**	66±9.1^a^	28±3.1	33±3.7	72±6.7^b^	38±2.6^b^	31±3.5	29±2.9	33±3.7
**HP (mg/g tissue)**								
Day 0	118.3±1	117.9±2.4	133±1.4	119±1.6	119.2±2.1	117.6±2.7	119.5±2	133±1.4
Day 15	133.6±0.6^a^	38.3±2.1	90.2±0.8^b^	87.6±2^b^	81.1±3^b^	78.5±1.6^b^	37.6±1.1	90.2±0.8^b^

Values are expressed as mean±SD from 6 rats/group.

a :significantly different from diabetic control group (PBS i.v) at *p*≤0.01,

b :significantly different from diabetic control group (topical saline) at *p*≤0.01.

**Figure 2:**
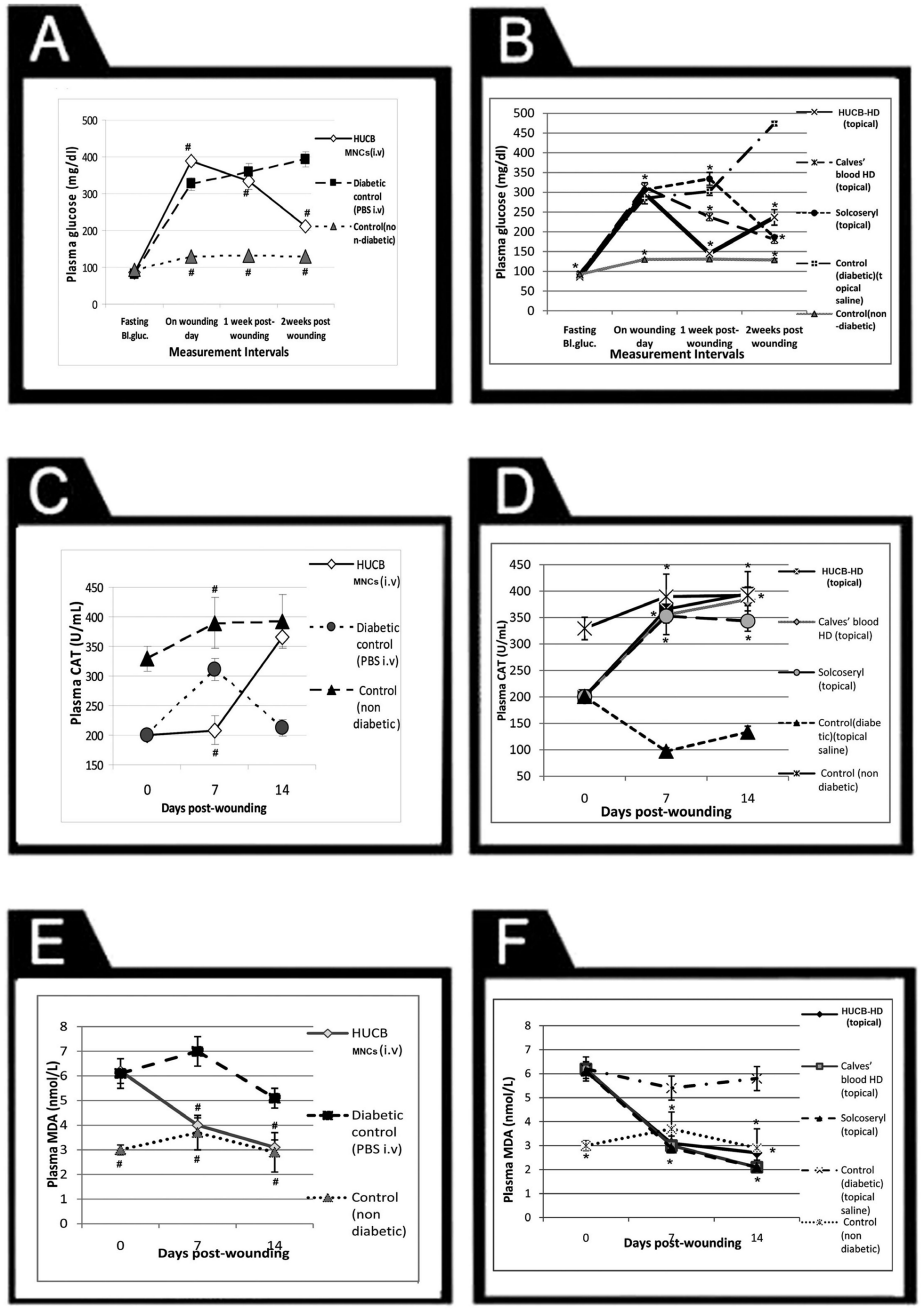
Plasma glucose (mg/dL), CAT (U/mL), and MDA (nmol/mL) levels in the different studied groups. Left column represents the 1^st^ experiment (i.v); effect of HUCB MNCs i.v and the 2^nd^ is represented in the right column (topical experiment); effect of HUCB-HD, calves' blood HD, and solcoseryl topical treatment. Plasma glucose (A, B) was measured while fasting, on the wounding day, and then on a weekly basis for 2 weeks. CAT (C, D) and MDA (E,F) were measured on the wounding day, on day 7, day14 post-wounding. Values are expressed as mean ± SD from 6 rats/group. # significantly different from diabetic control group (PBS i.v), at *p*≤0.001, * significantly different from diabetic control group (topical saline), at *p*≤0.001.

Administration of either HUCB MNCs (i.v) or HUCB-HD (topical) resulted in a significant (p≤0.01) antihyperglycemic effect that was evident from the first week onwards. Moreover, the decrease in blood glucose was significant on the second week in groups treated with calves' blood HD and solcoseryl compared to the diabetic untreated groups, which showed a progressive increase in the blood glucose level throughout the period of wound healing (**Figure 2A, B**).

Before treatment, on day 0, it was found that all diabetic groups showed oxidative stress markers represented by reduced antioxidant CAT enzyme level, elevated MDA, and reduced NO level as compared to normal non-diabetic control groups of both experiments. Treatment with HUCB MNCs resulted in elevation of CAT on day 7 post-wounding followed by a significant (p<0.001) increase on day 14 post-wounding (**Figure 2C**). Likewise, a significant increase in CAT level was observed in all HD-treated groups on days 7 and 14 post-wounding as compared to control diabetic groups (**Figure 2D**), but the extent of increase was higher in HUCB-HD, calves' blood HD, and better than solcoseryl, where the CAT level was significantly reversed to near normal and the significant increase was observed as early as day 7 compared to the HUCB MNCs. Analysis of MDA on day 7 and day 14 post-wounding showed a significant (p<0.001) decrease in MDA following treatment with HUCB MNCs and HUCB-HD compared to diabetic untreated control groups of both experiments (**Figure 2E, F**).

**Figure 3:**
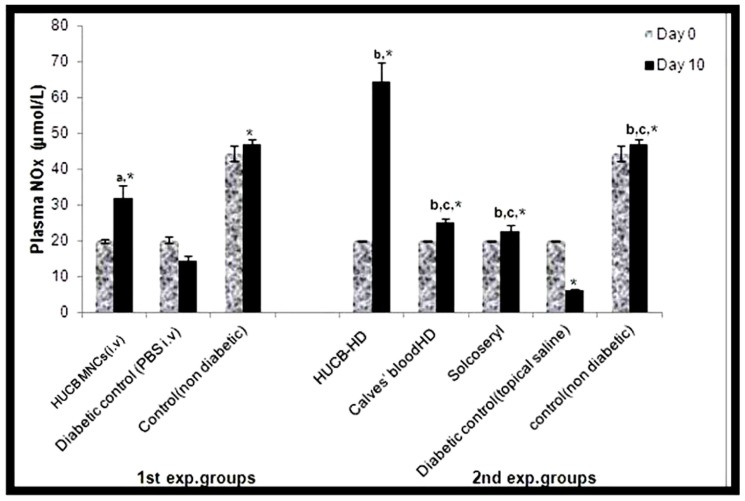
Effect of different therapeutic agents of both experiments on plasma NOx (µmol/L) measured on day 0 and day 10 post-wounding. Values are mean ± SD from 6 rats/group. a, b, c significantly different from diabetic control (PBS i.v), diabetic control (topical saline), and HUCB-HD, respectively, at *p*≤0.001. *Significant increase on day 10 compared to day 0 at *p*≤0.05.

NO level at day 10 (32.1±3.5 µmol/L) for the HUCB MNCs group was found to be 2-fold greater than the corresponding diabetic control (PBS i.v) group (14.6±1.3 µmol/L). Production of NO was elevated in solcoseryl and calves' blood HD-treated groups in response to treatment evidenced by high NO levels measured on day 10 post-wounding (22.6±1.8 and 25±1.1 µmol/L), respectively. Moreover, it was highly significantly (p≤0.001) increased in HUCB-HD-treated group (64.3±5.3 µmol/L) when compared to its diabetic (topical saline) control group (6.1±0.6 µmol/L) and approximately three-folds greater than its day 0 level (20±0.2 µmol/L) (**Figure 3**).

Induction of DM resulted in reduction of HP content (**Table 1**) in diabetic rats' skin granulation tissue as evidenced by low HP content measured on day 0 for all diabetic rats as compared to normal non-diabetic ones. After 15 days of injection with HUCB MNCs, a significant (p≤0.001) elevation in HP content was observed (133.6±0.6 mg/g tissue) which even exceeded its skin content in normal non-diabetic rats before ulcer induction (133±1.4 mg/g tissue). Although treatment with HUCB-HD, calves' blood HD, or solcoseryl resulted in significant (p≤0.001) elevation of HP content compared to the diabetic control group, the HP content attained by the HUCB-HD-treated group (87.6±1.9 mg/g tissue) was higher than the other two groups and very close to the content attained by the normal non-diabetic control group (90.2±0.8 mg/g tissue) after 15 days of treatment.

Meanwhile, a significant (p≤0.001) increase was observed in the dry and wet granulation tissue's weight in groups treated with HUCB MNCs, calves' blood HD and HUCB-HD in comparison to their corresponding diabetic control groups (**Table 1**).

**Figure 4:**
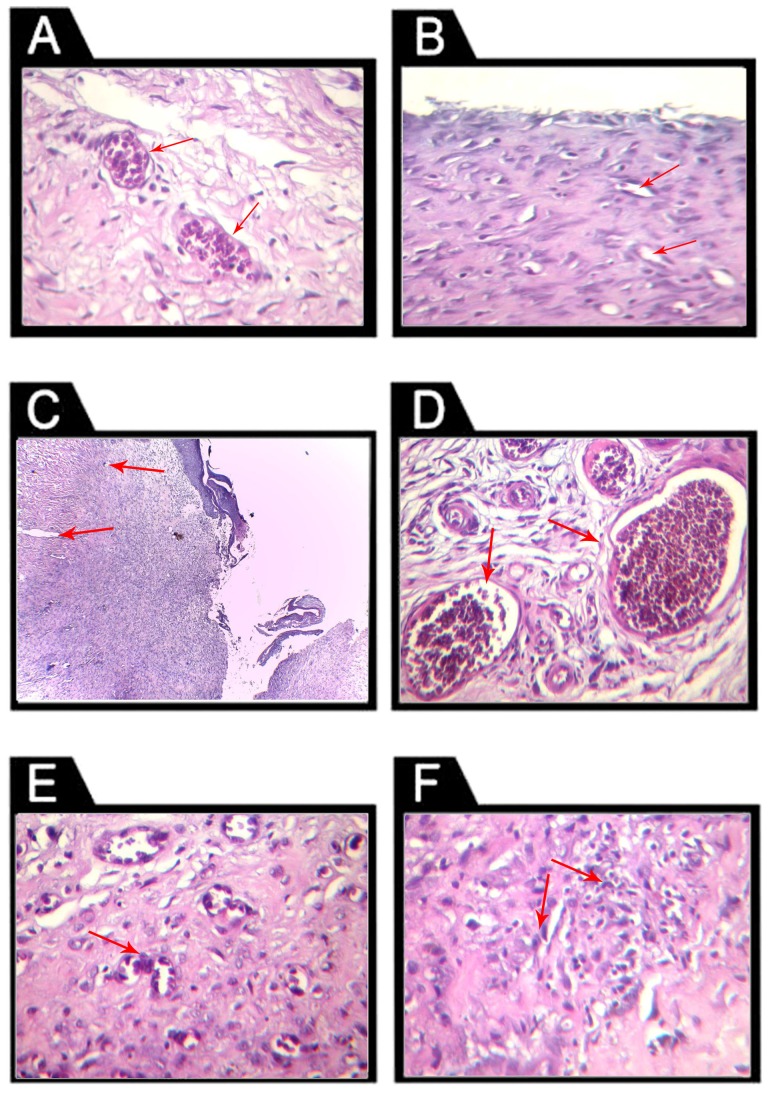
Representative photomicrographs of studied wound sections on day 14 post-wounding stained with hematoxylin-eosin stain (H&E). A: diabetic wounds treated with HUCB MNCs (i.v) showing marked vascular proliferation (arrows) and fibroplasia with increased re-epithelialization (×400). B: diabetic control wounds (PBS i.v) showing failure of wound closure at the upper area of the field with subepithelial exudates of neutrophils, blood vessels were immature (arrows) with minimal proliferation (×200). C: diabetic wound treated topically with solcoseryl showing mild angioplasia encountered, but blood vessels are not yet fully mature, with collapsed lumens. A marked fibro-collagenous reaction (arrows) is encountered and epithelial proliferation is minimal (×100). D: diabetic wound treated topically with HUCB-HD, where marked mature vascular proliferation (arrows) and fibroplasia were noticed, as well as adequate epithelization with subepithelial relatively high angiofibroplasia (×200). E: diabetic wound treated topically with calves' blood HD showing mature vascular proliferation (arrows) and moderate exudates of macrophages, surface epithelization with moderate inflammation, mostly of macrophages (×400). F: diabetic control (topical saline) wound showing failure of wound closure attempts at the upper area of the field, with subepithelial exudates of neutrophils, sustained high neutrophilic count with immature fibrous proliferation. Blood vessels were of a low count appearing irregular and immature (arrows) (×200).

Wounds histopathological changes on day 14 are presented in **Figure 4**. HUCB MNCs and HUCB-HD-treated wounds showed earlier wound contraction and earlier onset of epithelization than calves' blood HD and solcoseryl-treated wounds. This was evidenced by the presence of marked vascular proliferation and fibroplasia. However, diabetic control groups showed failure of wound closure attempts and little evidence of re-epithelialization.

## Discussion

Development of new treatment modalities to improve wound healing in diabetic patients is an essential and emerging field of investigation [32], as medical therapies for wound care are limited, the outcome of management is poor and there is uncertainty concerning optimal approaches to management.

Cell-based therapy is an attractive approach for the treatment of wounds with multiple impairments. Healing activity of stem cells is recognized for their ability to separate into the various component cells of injured tissues, as well as to discharge growth factors that may encourage the formation of new blood vessels [33].

Reduction in wound area, in the 1^st^ experiment, was faster in the MNCs-treated group compared to the diabetic and non-diabetic control groups. Similarly, in the 2^nd^ experiment, wound contraction was greater and more prominent in the HUCB-HD-treated group, followed by the calves' blood HD group and finally the solcoseryl group. Therefore, systemic administration of MNCs in the 1^st^ experiment or topical application of HUCB-HD in the 2^nd^ experiment significantly accelerated the rate of wound closure. Despite the comparative results displayed by HUCB MNCs and HUCB-HD, however HUCB-HD is superior for treatment as evidenced by showing an average healing time about 2.2 days shorter than the HUCB MNCs-treated group. These results agree with **Gurdol and colleagues** [34] who observed progressive reductions in wound areas following treatment of diabetic patients foot ulcers using hyperbaric oxygen. A study by **Hamed and colleagues** [35] investigated the effect of topical erythropoietin treatment on defective wound repair in diabetic rats skin. Erythropoietin-containing creams promoted skin wound repair in diabetic rats and the area of open wounds was significantly smaller than that of vehicle-treated wounds. In a human study of chronic non-healing wounds, it was shown that directly applied bone marrow-derived cells can lead to wound closure and possible tissue rebuilding [36].

HUCB MNCs were capable of normalizing blood glucose level in diabetic rats, an effect attributed to the generation of HUCB-derived insulin-producing cells which can be mediated through both fusion-dependent and independent mechanisms. This is in agreement with different *in vitro* and *in vivo* studies which clarified the capability of HUCB-derived cells to generate insulin-producing cells. **Hitherto**, one study carried out by **Pessina and colleagues**, has investigated the expression of endocrine pancreatic progenitor markers in HUCB cells [37]. All the HD compounds administered were also shown to improve blood glucose levels in diabetic rats. However, HUCB-HD was capable of even normalizing blood glucose levels. This may be reclaimed to the clear insulin-like effects of HD compounds on glucose uptake and utilization which have been demonstrated *in vitro* [38] and in different pharmacodynamic studies following the use of a deproteinized HD. Improvement of cellular glucose uptake is based on direct activation of glucose transporters located in the cell membrane. This activation was demonstrated to reach an extent comparable to that of insulin [39]. Moreover, this insulin-like activity of the HD can be explained in the terms of inositolphosphooligosaccharides (IPOs) compounds, which activate glucose transporters, with a direct influence on the activities of some enzymes involved in glucose metabolic pathways [40].

Blood glucose levels measured were found to be strongly and inversely related to body weight of rats, where treatment with HUCB MNCs and HUCB-HD resulted in an early and steady increase in body weights (data not shown) till the end of the experiment. In contrast, diabetic control groups exhibited an overall persistent loss of body weight. These results are consistent with the results of [41], [42]

Treatment of diabetic wounds with HUCB-derived MNCs, prepared HD of both HUCB and local buffalo calves' blood resulted in alleviation of oxidative stress in wounded STZ-induced diabetic rats. These results are consistent with the results of **Nwanjo and colleagues** [41] and can be explained by the ability of treatment agents to improve activities of the hepatic anti-oxidant enzymes; consequently scavenging toxic reactive oxygen free radicals which are responsible for tissue damage in STZ-induced DM, as well as inhibition of lipid peroxidation and thereby tissues and organs oxidative damage.

Ongoing experimental and clinical wound healing studies have presented NO as a critical mediator of tissue repair [43]. NO is also the most effective antioxidant being a scavenger of superoxide. Moreover, impaired wound healing in diabetics has been associated with reduced NO synthesis. Thus, high level of bioavailable NO in wounds is important for enhancing the healing process [44]. In our study we aimed to determine total NO_2_^−^/NO_3_^−^ level, which represent the stable oxidation product of NO as an indicator of NO production in plasma, and to evaluate its relation to healing during foot ulcers' treatment.

Evidence shows that decrease of NO bioavailability might be related to elevation of endogenous nitric oxide synthase (NOS) inhibitors [45]. In diabetes-induced hyperglycemia, there is an increased metabolism of glucose to sorbitol via the polyol pathway. Increased activity of the enzyme aldose reductase requires and may deplete cellular NADPH, which is also a required cofactor for NOS [46]. It is possible that both mechanisms occur during diabetic wound healing and results in drastically reduced NO production [47]. Therefore, treatment with HUCB-derived MNCs and HUCB-HD might have resulted in decreasing NOS inhibitors levels or increasing the availability of cellular NADPH. These effects are probably secondary to glycemic control improvement as well as their antioxidant properties [34]. Nevertheless, the elevation in NO level following treatment with HUCB-HD was remarkable and exceeded that attained by the normal control group. HUCB-HD would be able to elevate NO levels efficiently to an optimum level, contributing to the healing process by reversing the impaired healing condition associated with DM and restoring wound NO levels towards more normal values.

Collagen plays a central role in wound healing, being the principal component of connective tissue providing a structural framework for tissue regeneration [16]. Measurement of HP could be used as an index for collagen turn over [48]. Hence, its estimation in the granulation tissue may throw light on maturation and the healing process [49]. In our study, granulation tissue HP level was significantly increased in both HUCB MNCs group and HUCB-HD group following treatment. However, the substantial rise in HP following MNCs treatment may be attributed to the paracrine effect of stem cells that can promote the wound healing progress [7]. An *in vitro* study indicated that collagen synthesis and levels of bFGF and VEGF were much higher in bone marrow stem cells than those in dermal fibroblasts, so the ability of adult stem cells to alter tissue microenvironment via secretion of soluble factors may contribute to tissue repair even more than their multipotential differentiation [50]. Decreased HP content measured in the untreated diabetic rats' wounds parallels decreased weight of granulation tissue in this study, while the observed increase in wet and dry weights of granulation tissue in groups treated with HUCB MNCs and HUCB-HD reflected higher protein content and better healing [48]. This was much confirmed through the histopathological examination of skin tissue samples, where HUCB MNCs were found to decrease wound inflammatory cell infiltration, accelerated new blood vessels formation “angiogenesis”, and granulation tissue. This effect is related to the self-renewal and multipotency properties, which stem cells have. Adult stem cells can produce differentiated skin cells and a previous study have found that MSCs, isolated from different tissues including UCB, can contribute to skin reconstitution in cutaneous wounds [51]. The potential of MSCs to differentiate into skin tissue cells has been described [52]. In a study by Korean researchers, transplanting HUCB-derived endothelial progenitor cells (EPCs) resulted in a significant acceleration of wound closure in diabetic mouse models [53]. Adult stem cells can also modulate immune and inflammatory responses to promote wound healing; the transplantation of autologous and allogenic MSCs on the surface of deep burn wounds in rats decreased inflammatory cell infiltration into the wound, and accelerated new blood vessels and granulation tissue formation [54]. The marked vascular proliferation and fibroplasia observed in tissues after 14 days of treatment confirmed near closure. A variety of CB populations have been used including MNCs fractions, with significant beneficial effects. Undoubtedly, the beneficial effects could be attributed to induction of angiogenesis due to the release of a variety of angiogenic and growth factors [55]. Our results concerning HUCB-HD on day 14 post-wounding revealed that the granulation tissue in healed wound contained comparatively few inflammatory cells, more collagen, early efficient epithelization and marked mature vascular proliferation. This is because our HD and fractions isolated from it – consisting exclusively of low molecular weight compounds stimulate the proliferation of coronary endothelial cells which is not a trivial nutritive effect. The ability of endothelial cells to proliferate and to differentiate is a prerequisite of angiogenesis. Thus, agents stimulating the proliferation of EC are of special interest [56]. In addition, HD might strengthen the angiogenic effect of EGF on EC consisting of a proliferation-promoting and a chemoattractive activity, the latter demonstrated in wound fluids [56]. These synergisms would result in faster neovascularization probably accompanied by an improved fibroplasia during formation of granulation tissue. And this synergistic effect is believed to explain the therapeutic efficacy of HD in cases of impaired wound healing, ulcers, burns and occlusive diseases documented by several clinical and experimental studies [57], [58]. The present study has also shown that, in rats, HD compounds reduced the inflammatory phase duration in wound healing. This finding is consistent with the observation of **Dri and colleagues** that the HD interacts with polymorphnuclear leucocytes (PMNL) in a dual fashion; higher HD concentrations stimulate PMNL activity, whereas lower concentrations inhibit such activity [59]. HD improves the anabolic phase of healing by providing the wound with sufficient immediately utilizable and necessary low molecular weight starting materials and energy carriers for repair and energy metabolism [60]. We hypothesize that the reason for the enhanced vascularization and the superior positive variables encountered in wounds of HUCB-HD-treated group may be related to its composition. Therefore, a sample of this novel type of HD and another sample of the prepared calves' blood HD were taken for HPLC-ESI-MS analysis in order to find out if there is a difference in the composition. Analysis of the HUCB-HD sample revealed glutamate to be the predominant free amino acid, which is not the case in the Calves' blood HD (**Figure S1**).

Glutamine is a non-essential amino acid that can become a “conditionally essential” amino acid in certain circumstances, including tissue injury/repair [61]. It is used by the inflammatory cells within the wound for proliferation and as a source of energy [62]. Fibroblasts use glutamine for these same purposes, as well as for protein and nucleic acid synthesis. Glutamate is also a migration-promoting factor of neutrophils to the wound site. Rapid migration of neutrophils to the wound site is a prerequisite to the wound healing process. According to a previous study by **Gupta and Chattopadhyay** on placental extract, it was shown that glutamate at an optimum concentration induced phenotypic neutrophil chemotaxis as well as neutrophil chemokinesis [63]. Glutamate-induced chemotaxis was accompanied by polarization of the actin cytoskeleton and by polymerization of F-actin. Moreover, glutamine plays a key role in the immune system [64], so deficiency in this nutrient can significantly slow the healing process. This explains the probable superior wound healing potential of the HUCB-HD over the calves' blood-HD.

Additionally, the present study recorded a relative decrease in platelet count (data not shown) of STZ-induced diabetic control rats similar to that found in human DM, which was corrected only in HUCB MNCs and HUCB-HD-treated groups (data not shown). This finding agree with **Shi and colleagues** who reported a promotion of hematopoietic recovery following treatment of combined radiation and wound injury with a systemic infusion of dermal multipotent cells. They indicated that stem cells through an indirect mechanism could produce a series of cytokines and extracellular matrix molecules, including IL-3, IL-6, IL-15, VEGF, PDGF, HGF, TGF-β, ICAM-1, VCAM-1, and fibronectin, which were closely related to hematopoiesis and wound healing [65].

Increase in platelets count in case of HUCB-HD may be attributed to the indirect mechanism of decreasing the platelets turnover, increasing their survival time or decreasing their aggregability due to improving disturbance in carbohydrate metabolism associated with diabetes. Disturbed carbohydrate metabolism was suggested to be directly or indirectly causing increased platelet aggregation [66] an effect resulting from the insulin-like effect of the HD mentioned previously.

## Conclusions

Our results revealed that systemic administration of HUCB-derived MNCs or topical treatment with calves' blood HD and the novel HUCB-derived HD can improve the impaired healing of diabetic wounds as manifested by shortened epithelialization time and rapid wound contraction. These healing promoting effects can be explained on the basis of several mechanisms; antihyperglycemic mechanisms, antioxidant mechanisms, reduction of lipid peroxidation product (MDA) and increase of NO production; a critical mediator of tissue repair, in addition to increasing HP content in the granulation tissue. However, the HUCB-derived HD has turned out to be the best among all treatment agents due to the fastest wound closure and shortest healing time achieved. This could be probably explained by the multifactorial nature of this approach that covers the whole multiphase complex process of wound healing. Results of this study suggest that topical application of HUCB–derived HD should be studied in human diabetic foot ulcers and the mechanism of its action should be further investigated.

## Supporting Information

Figure S1 **Mass spectra of two HD samples.** Mass spectra of two HD samples derived from human umbilical cord blood (a) and Calves's blood (b), respectively are shown. Both samples were subjected to LC-ESI-MS/MS protein analysis. Amino acid content of each type of HD is represented as peaks at their specific m/z ratio, with a relative intensity corresponding to the amount of each amino acid in the sample.
(TIF)
